# The triglyceride glucose index was U-shape associated with all-cause mortality in population with cardiovascular diseases

**DOI:** 10.1186/s13098-023-01153-3

**Published:** 2023-09-07

**Authors:** Haiyu Li, Yaohui Jiang, Xin Su, Zhe Meng

**Affiliations:** Department Cardiology, The First Affiliated Hospital of Zhengzhou University, Zhengzhou, China

**Keywords:** Triglyceride glucose index, All-cause mortality, Cardiovascular mortality, Insulin resistance, NHANES

## Abstract

**Background:**

The triglyceride and glucose (TyG) index has been considered a simple surrogate marker of insulin resistance, related to a high risk of mortality. However, few studies have investigated the specific relationship between the TyG index and all-cause mortality among population with cardiovascular diseases.

**Methods:**

2,072 participants with cardiovascular diseases were enrolled from the National Health and Nutrition Examination Survey (NHANES) 1999–2014. The TyG index was calculated as log [fasting triglycerides (mg/dL) x fasting glucose (mg/dL)/2]. Outcomes were all-cause mortality and cardiovascular mortality. The baseline levels of TyG associated with the risk of mortality were evaluated on a continuous scale (restricted cubic splines) and by a priori defined quantile categories with Cox regression models.

**Results:**

After a follow-up of 16.8 years, 791 all-cause deaths and 184 cardiovascular deaths occurred. Restricted cubic splines showed that the association between levels of TyG index and the risk of all-cause mortality was non-linear (p < 0.001) and the TyG index associated with the lowest risk of all-cause mortality ranges 8.83 to 9.06 in individuals with cardiovascular diseases. Compared with the reference quartile of 8.84 ~ 9.29, the multivariate-adjusted hazards ratios and 95% confidence intervals were 1.40 (1.13–1.74; p = 0.002) in the lowest quartile and 1.08 (0.88, 1.32; p = 0.475) in the highest quartile for all-cause mortality. However, TyG was not associated with cardiovascular mortality.

**Conclusions:**

TyG index was U-shape associated with the risk of all-cause mortality in participants with cardiovascular diseases and the level associated with the lowest risk ranged 8.83 to 9.06.

## Introduction

The triglyceride glucose (TyG) index has been suggested as a surrogate marker of insulin resistance, a pathological condition characterized by poor insulin sensitivity in the peripheral tissues [1, 2].

Cardiovascular disease (CVD) is a leading cause of death worldwide. Several studies have suggested that the TyG index is associated with arterial stiffness [3], coronary stenosis [4] or calcification [5], contributing to a high risk of cardiovascular diseases [6]. Besides, TyG index was an independent predictor of mortality in general population [7], including type 2 diabetes [8], stroke [9] and acute myocardial infarction [10]. Besides, a prospective cohort study confirmed that the TyG index was significantly associated with future cardiovascular mortality, myocardial infarction, stroke, and type 2 diabetes [11]. However, the specific relationship between TyG index and the long-term mortality in cardiovascular diseases was not investigated.

Therefore, our study evaluated the association between the TyG index and all-cause and cardiovascular mortality in population with cardiovascular diseases.

## Materials and methods

### Study population

**Fig. 1:**
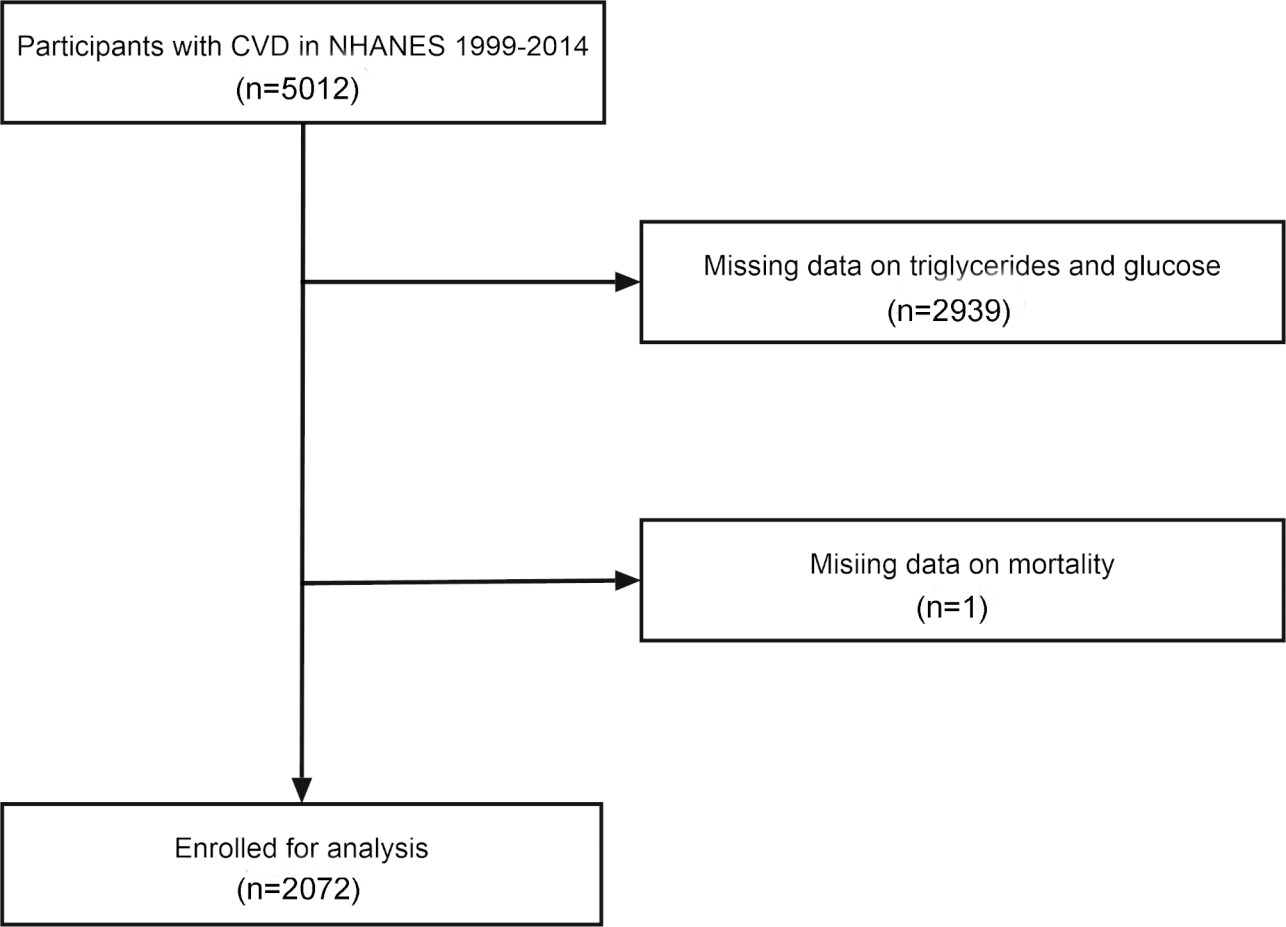
The flow chart of participants selection

The retrospective cohort study included individuals from the National Health and Nutrition Examination Survey (NHANES) between the periods of 1999–2014, a nationwide survey conducted by the National Center for Health Statistics (NCHS) in United States. The cardiovascular disease (CVD) was defined as self-reported congestive heart failure, coronary heart disease, angina pectoris, heart attack and stroke. The participant was recorded as having CVD if she/he answered “yes” to the following question: “Has a doctor or other health professional ever told you that you had congestive heart failure/coronary heart disease/angina pectoris/stroke?” in a validated questionnaire (https://wwwn.cdc.gov/Nchs/Nhanes/2013-2014/MCQ_H.htm). Among 5,012 participants with CVD, we excluded 2,939 participants with missing data on fasting triglycerides and glucose, as well as one individual with missing mortality. In total, 2,072 participants were enrolled in our study. Figure 1 depicted the selection process. All participants provided written informed consent and the protocol was approved by NCHS Research Ethics Review Board (Protocol #98 − 12, Protocol #2005-06, and Protocol #2011-17).

### Exposure variable and outcomes

TyG index was calculated as ln[fasting triglycerides (mg/dL) x fasting glucose (mg/dL)/2]. Both the concentrations of triglycerides and glucose were measured enzymatically using Roche Modular P chemistry analyzer. Serum triglyceride concentration was measured using the Roche Modular P and Roche Cobas 6000 chemistry analyzers. Fasting plasma glucose was measured by the hexokinase-mediated reaction using Roche/Hitachi Cobas C 501 chemistry analyzer. The primary outcome was all-cause mortality while the secondary outcome was cardiovascular mortality. Mortality status was obtained by linkage to the National Death Index by 31 December 2015. Cardiovascular disease was defined as ICD-10 codes I00-I09, I11, I13, I20-I51, I60-I69 and I70-78.

### Covariate collection

Information on age, sex, race, education level, smoking status, drinking status, physical activity, and comorbidities were collected by using standardized questionnaires. The height and weight of each participant were obtained from the physical examinations. Fasting serum low-density lipoprotein (LDL) and high-density lipoprotein (HDL) were measured enzymatically using Roche Modular P chemistry analyzer. Body mass index (BMI, kg/m2) was calculated as weight divided by height squared. Race/ethnicity was classified as non-Hispanic white, non-Hispanic black, Mexican American or other race. Education level was categorized as less than high school, high school or equivalent and college or above. Smoking status were defined as current, past and never. Physical activity was categorized as vigorous, moderate and inactive. Hypertension was defined as the self-report hypertension, or systolic blood pressure ≥ 140 mmHg, or diastolic blood pressure ≥ 90 mmHg, or taking antihypertensive drugs. Diabetes was defined as a history of diabetes or fasting glucose > 7 mmol/L or glycated hemoglobin A1c > 6.5% or use of hypoglycemic medication. Multiple imputation using predictive mean matching was performed for covariates with missing values. Predictor variables included all covariates from the main analysis, survey weight, and unique groupings of sampling strata and primary sampling unit. Ten imputed datasets were generated and pooled to obtain the overall result [12].

### Statistical analysis

Data are presented as mean ± standard deviation or number (proportions). Differences among different TyG index groups were explored by one-way analysis of variance and chi-square test. Associations between TyG index and the risk of all-cause and cardiovascular mortality were estimated by multivariate Cox regression models with 95% confidence intervals. The selection of confounding variables was based on the significant difference among TyG quartiles and clinical relevance of all-cause and cardiovascular mortality. Model 1 was unadjusted. Model 2 was adjusted for age, and gender. Model 3 was adjusted for age, gender, race, education, BMI, smoking, drinking, physical activity, hypertension, diabetes, HDL and LDL. The specific associations between levels of TyG index and all-cause mortality was evaluated on a continuous scale with restricted cubic spline. All analysis were performed using the statistical package R version 3.6. All P values were two-sided with a significance level of < 0.05.

## Results

The present study included 2,072 participants with an average of 67.1 years old and 1174 (56.7%) male individuals. The baseline characteristics of the study population according to TyG quartile were shown in the Table 1. The ranges of TyG index for quartile 1–4 were < 8.43, 8.43 ~ 8.83, 8.84 ~ 9.29, and > 9.29 respectively. The highest quartile was more likely to be younger and had more percentage of smokers and diabetes. Besides, the level of HDL was lower with the increment of TyG quartiles.

**Table 1:** The characteristics of participants according to TyG index

Variable	Overall N = 2072	Q1 N = 518	Q2 N = 518	Q3 N = 518	Q4 N = 518	P value
Male, %	1174 (56.7)	313 (60.4)	281(54.2)	274 (52.9)	306 (59.1)	0.038
Age, years	67.1 (12.9)	67.3 (14.4)	67.9 (12.8)	67.2 (12.3)	66.1 (12.1)	0.173
Race, %						< 0.001
Non-Hispanic white	1214 (58.6)	287 (55.4)	302 (58.3)	315 (60.8)	310 (59.8)	
Non-Hispanic black	403 (19.4)	144 (27.8)	98 (18.9)	90 (17.4)	71 (13.7)	
Mexican American	251 (12.1)	36 (6.9)	51 (9.8)	75 (14.5)	89 (17.2)	
Others	204 (9.8)	51 (9.8)	67 (12.9)	38 (7.3)	48 (9.3)	
Education, %						0.001
Less than high school	803 (38.9)	183(35.5)	201 (38.9)	194 (37.6)	225 (43.9)	
High school or equivalent	476 (23.1)	104 (20.2)	120 (23.2)	119 (23.1)	133 (25.9)	
College or above	783 (38.0)	229 (44.4)	196 (37.9)	203 (39.3)	155 (30.2)	
BMI, kg/m2	29.8 (6.7)	27.7 (6.1)	28.9 (6.5)	30.9 (6.9)	31.8 (6.5)	< 0.001
Smoking, %						0.232
Never	827 (66.5)	210 (67.7)	218 (66.7)	212 (67.9)	187 (63.4)	
Past	60 (4.8)	16 (5.2)	12 (3.7)	21 (6.7)	11 (3.7)	
Current	357 (28.7)	84 (27.1)	97 (29.7)	79 (25.3)	97 (32.9)	
Drinking, %	363 (56.5)	90 (64.3)	85 (54.1)	102 (55.7)	86 (53.1)	0.202
Activity, %						0.859
Inactive	156 (17.4)	44 (19.0)	39 (17.5)	36 (15.9)	37 (17.3)	
Moderate	498 (55.7)	126 (54.5)	123 (55.2)	123 (54.4)	126 (58.9)	
Vigorous	240 (26.8)	61 (26.4)	61 (27.4)	67 (29.6)	51 (23.8)	
Hypertension, %	676 (34.2)	148 (29.7)	153 (30.8)	196 (40.0)	179 (36.3)	0.001
Diabetes, %	798 (38.5)	99 (19.1)	142 (27.4)	210 (40.5)	347 (67.0)	< 0.001
White blood cell, 1000/ul	7.24 (2.17)	7.21 (2.18)	7.23 (2.21)	7.28 (2.11)	7.20 (2.15)	0.654
Glucose, mg/dL	120.9 (44.7)	99.6 (13.6)	107.0 (20.1)	118.3 (29.9)	158.8 (66.5)	< 0.001
Triglycerides, mg/dL	151.3 (135.8)	68.9 (15.8)	108.4 (20.5)	152.6 (35.3)	275.1 (219.0)	< 0.001
HDL, mg/dL	50.7 (15.9)	60.3 (18.7)	52.6 (14.7)	47.6 (12.1)	42.3 (11.6)	< 0.001
LDL, mg/dL	106.1 (38.3)	97.8 (33.9)	106.7 (36.3)	110.9 (38.4)	109.7 (43.4)	< 0.001

Data are presented as mean (SD) or n (%). Q1: TyG < 8.43; Q2: 8.43 ~ 8.83; Q3:8.84 ~ 9.29; Q4: >9.29. BMI, body mass index; HDL, high-density lipoprotein; LDL, low-density lipoprotein

**Fig. 2:**
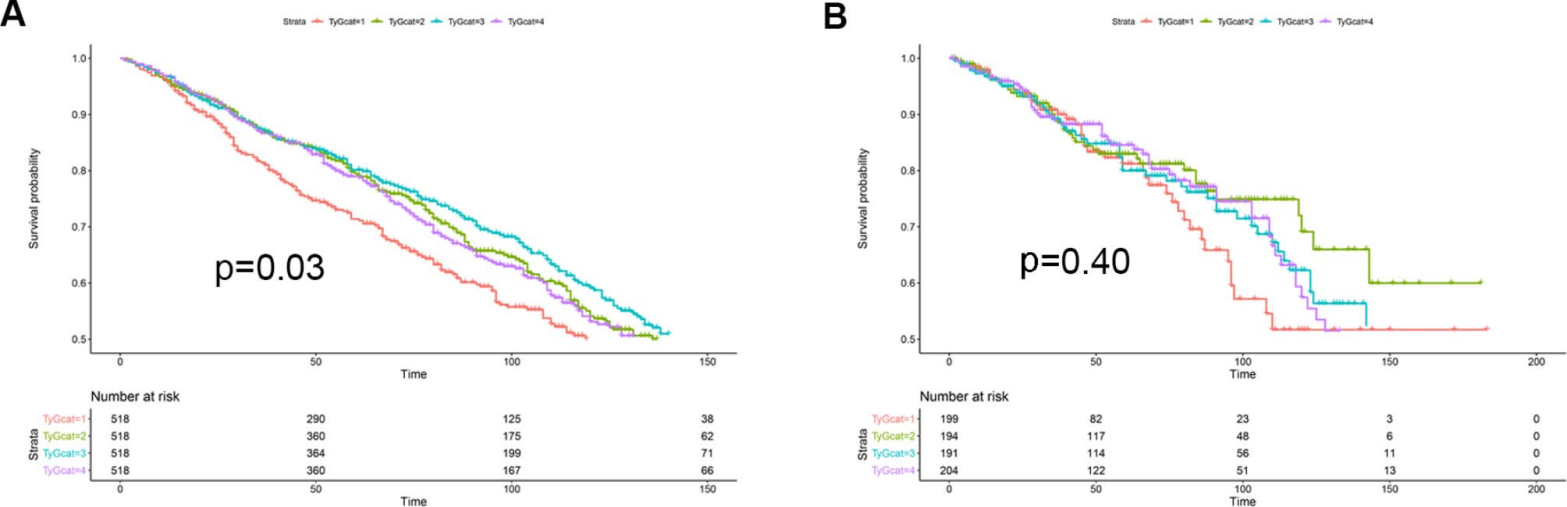
The Kaplan-Meier analysis of the prognostic effect of TyG index on all-cause mortality (A) and cardiovascular mortality (B). TyGcat represented the quartiles of TyG index. The unit of time was month

During the 16.8-year of follow-up, 791 all-cause deaths and 184 cardiovascular deaths occurred. As shown in Fig. 2, Kaplan–Meier analysis suggested that the lowest and highest TyG level were associated with a higher all-cause mortality, but not with cardiovascular mortality.

**Fig. 3:**
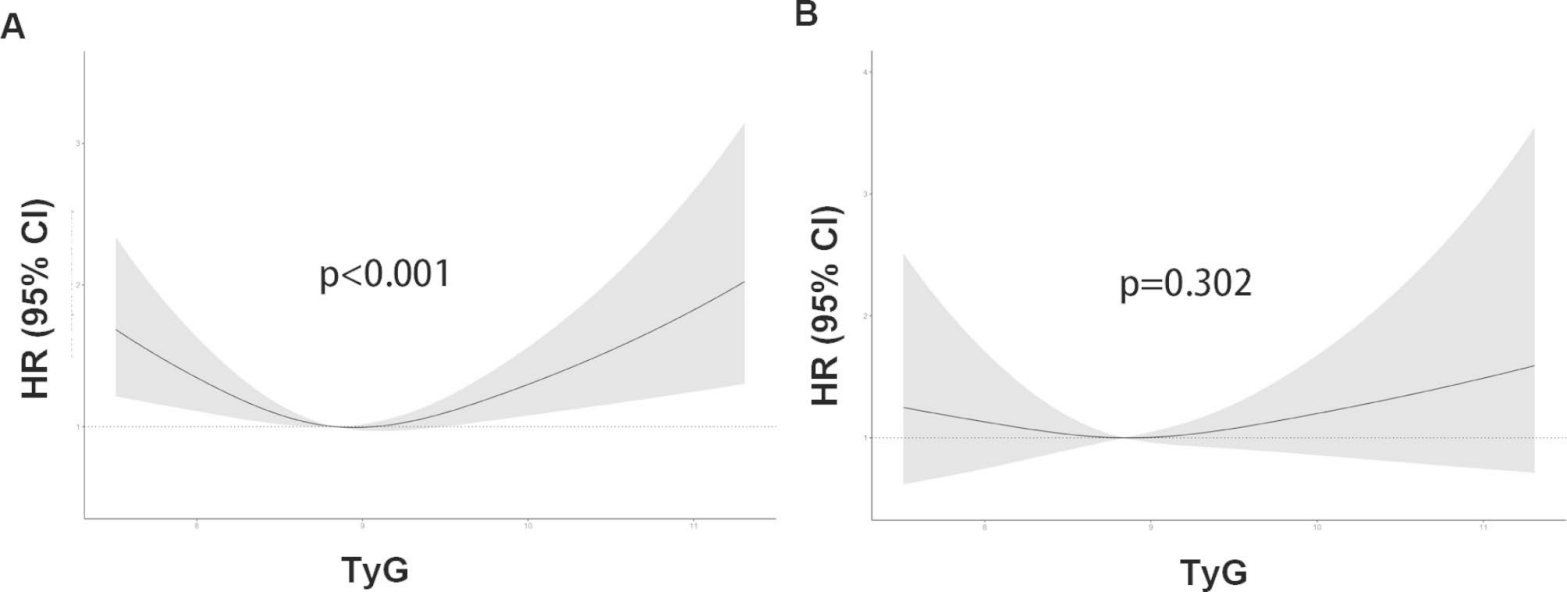
The restricted cubic regression between TyG index with all-cause mortality (A) and cardiovascular mortality (B) in fully adjusted model

Restricted cubic spline regression suggested that TyG index was U-shape associated with the risk of all-cause mortality (p for nonlinearity < 0.001) (Fig. 3). And the TyG index associated with the lowest risk of all-cause mortality ranged 8.83 to 9.06 in individuals with cardiovascular diseases.

Therefore, the 3rd quartile was set as the reference group. As shown in Table 2, three Cox regression models were constructed after adjusting for demographics, lifestyles and medical histories. When compared with the reference quartile of 8.84 ~ 9.29, the multivariable-adjusted hazard ratios for all-cause mortality were 1.33 (1.09–1.62; p = 0.005) in Model 1, 1.26 (1.04–1.54; p = 0.021) in Model 2 and 1.40 (1.13–1.74; p = 0.032) in Model 3 for the lowest quartile of < 8.43. The highest quartile was not associated with an increased risk of all-cause mortality in the fully-adjusted model (hazard ratio 1.08 [0.88, 1.32]; p = 0.475). Besides, we did not observe a significant association between TyG index with cardiovascular mortality.

**Table 2:** Association of TyG index with all-cause and cardiovascular mortality

	Cases	N	Model 1		Model 2		Model 3	
			h (95% CI)	P	HR (95% CI)	P	HR (95% CI)	P
**All causes**								
Q1	192	518	1.33 [1.09, 1.62]	**0.005**	1.26 [1.04, 1.54]	**0.021**	1.40 [1.13, 1.74]	**0.002**
Q2	200	518	1.07 [0.87, 1.30]	0.532	0.99 [0.81, 1.21]	0.940	1.06 [0.86, 1.30]	0.579
Q3	194	518	Ref	-	Ref	-	Ref	-
Q4	205	518	1.14 [0.94, 1.39]	0.196	1.23 [1.01, 1.50]	**0.037**	1.08 [0.88, 1.32]	0.475
Continuous	791	2072	1.00 [0.90, 1.11]	0.943	1.13 [1.01, 1.27]	**0.032**	1.03 [0.89, 1.18]	0.724
**Cardiovascular**								
Q1	38	194	1.18 [0.78, 1.78]	0.444	1.15 [0.76, 1.75]	0.508	1.10 [0.70, 1.72]	0.684
Q2	41	199	0.81 [0.53, 1.23]	0.323	0.81 [0.53, 1.24]	0.334	0.85 [0.55, 1.31]	0.462
Q3	50	191	Ref	-	Ref	-	Ref	-
Q4	55	204	1.04 [0.71, 1.53]	0.841	1.07 [0.72, 1.57]	0.742	1.09 [0.72, 1.66]	0.683
Continuous	184	788	1.02 [0.83, 1.26]	0.821	1.06 [0.86, 1.31]	0.581	1.08 [0.84, 1.40]	0.556

Model1: not adjusted

Model2: adjusted for gender and age

Model3: adjusted for gender, age, race, education, BMI, smoking, drinking, activity, hypertension, diabetes, LDL and HDL.

HR, hazard ratio; CI, confidence interval

## Discussion

In this study, we found that a higher TyG index or a lower TyG index was independently associated with increased risk of all-cause mortality among population with cardiovascular diseases. And the TyG index with the lowest risk of all-cause mortality ranged 8.83 to 9.06. These results confirmed that TyG index could be a reference value and a predictor in clinical practice.

A magnitude of publications reported that TyG increased the risk cardiovascular diseases [6, 10, 13]. Our results also showed that a higher TyG index above 8.98 increased the risk of all-cause mortality in individuals with CVD. This could be explained that participants with high TyG index had more percentage of hypertension, and diabetes, contributing to increased mortality. Most importantly, IR was one of the explanations for this association [14]. IR can induce glucose metabolism imbalance, which in turn triggers inflammation and oxidative stress. IR can induce an increased production of glycosylated products and free radicals, leading to nitric oxide (NO) inactivation. Besides, in addition to its role in hyperglycaemia, IR plays an important role in hyperlipidaemia [15].

Besides, we also found that a lower TyG index was related to higher risk of mortality, which may be related to a poor nutrition status. Xia et al. found that lower triglycerides were associated with chronic illness [16]. The TyG level associated with the lowest risk of mortality was 8.98. Most importantly, our results indicated that lower levels of glucose or triglycerides can lead to poorer prognosis. This makes a strong case for target ranges for triglycerides and glucose rather than target levels.

Many studies found that TyG index was an independent predictor for adverse cardiovascular events in both nondiabetic and diabetic subjects [17, 18]. It may be related to IR, which not only leads to the development of CVD in both the general population and diabetic patients but also predicts the cardiovascular prognosis of patients with CVD [19]. A prospective cohort study found that the TyG index was significantly associated with future cardiovascular mortality, myocardial infarction, stroke, and type 2 diabetes, suggesting that insulin resistance played a promoting role in the pathogenesis of cardiovascular and prognosis [11]. However, our study didn’t observe a significant association between TyG index and cardiovascular mortality. The effect of triglycerides or glucose on cardiovascular events could be eliminated by lipid-lowering drugs and hypoglycemic drugs in cardiovascular diseases. In supporting with our study, Laura et al. did not find an association between the TyG index and CVD in subjects with T2DM or hypertension at baseline [6]. Therefore, the application of the TyG index in CVD patients can be affected by hyperlipidaemia and diabetes [2]. Besides, the measurement of the TyG index at baseline alone does not reflect the longitudinal association between the TyG index and CVD risk over time [20]. Finally, the unrelated link may be due to lack of statistic power.

Our study has some limitations. Firstly, data on triglycerides and glucose were only collected once at baseline, and it was unclear whether TyG changes over time could affect the association with mortality. Secondly, the presence of CVD was self-reported based on questionnaires. Thirdly, the levels of triglyceride and glucose could be influenced by the prescribed medications, which was not reported in our study. Finally, the possibility of residual confounding existed since this is an observational study.

## Conclusions

We found a U-shape association between the TyG index and the risk of all-cause mortality among population with CVD. And the TyG index associated with the lowest risk of all-cause mortality ranged 8.83 to 9.06.

## Data Availability

The original data can be obtained from NHANES (https://www.cdc.gov/nchs/nhanes/index.htm).
